# The complete mitochondrial genome of *Boccardiella hamata* (Annelida: Polychaeta: Spionida)

**DOI:** 10.1080/23802359.2021.1964395

**Published:** 2021-08-13

**Authors:** Geon Hyeok Lee, Ha-Eun Lee, Gi-Sik Min

**Affiliations:** Department of Biological Sciences, Inha University, Incheon, Korea

**Keywords:** Complete mitogenome, Polychaeta, Spionida, *Boccardiella hamata*

## Abstract

In this study, the complete mitogenome sequence of Korean *Boccardiella hamata* was determined. This is the first complete mitogenome in the order Spionida. The complete mitogenome of *B. hamata* is 17,561 bp in length with 12 protein-coding genes (*atp8* gene absent), 23 transfer RNAs, 2 ribosomal RNAs, and 1 control region. Interestingly, the gene arrangement of the 12 PCGs of *B. hamata* is unique, which is very different from that of the other polychaetes currently known. The phylogenetic tree supported the traditional taxonomic position of Spionidae within subclass Sedentaria.

The family Spionidae Grube, 1850 is one of the largest groups of polychaetous annelids that commonly occur in a wide variety of habitats from intertidal to deep-sea and more than 500 nominal species of about 35 genera are currently known (Radashevsky 2012). Traditionally, Spionidae is placed in the subclass Sedentaria, which was named after their life habits and the development of the anterior part of the body (Audouin and Milne Edwards 1834; Fauchald 1977). This is supported in the recent polychaete phylogenetic studies using molecular data (Struck et al. 2015; Weigert and Bleidorn 2016). *Boccardiella hamata* (Webster 1879) , a relatively widespread species based on current knowledge, is distributed in temperate waters over the northern hemisphere (Kerckhof and Faasse 2014). In this study, the complete mitogenome sequence of Korean *B. hamata* was determined and is the first complete mitogenome in the order Spionida *sensu* (Rouse and Fauchald 1997).

The adult specimens of *B. hamata* were collected from the mud in crevices between the shells of oysters and their adherent substrates, attached to rocks in intertidal areas at Eurwang-dong, Incheon, South Korea (37°24'59N, 126°24'55E). The voucher specimen was deposited at the National Institute of Biological Resources in South Korea (deposit number: NIBRIV0000886083; Url: https://www.nibr.go.kr; Contact person: Min Seock Do, viper@korea.kr). The mitochondria from a live individual were isolated using Qproteome Mitochondria Isolation Kit (Qiagen, Hilden, Germany). The mitochondrial DNA (mtDNA) was extracted from the mitochondria using a LaboPass Tissue Mini (Cosmo GENETECH, Seoul, South Korea). Whole-genome amplification (WGA) of extracted mtDNA was performed using REPLI-g Mitochondrial DNA Kit (Qiagen, Hilden, Germany) according to the manufacturer’s instruction. The amplified mtDNA was sequenced using Illumina HiSeq 4000 (Macrogen, Seoul, Korea), and assembled by Geneious 8.1.9 (Biomatters Auckland New Zealand) and NovoPlasty v. 2.7.1 (Dierckxsens et al. 2017). The complete mitogenome was annotated with the MITOS Webserver (Bernt et al. 2013). The extracted mitochondrial DNA was deposited in the DNA collection at the National Institute of Biological Resources in South Korea (deposit number: NIBRGR0000627865). A maximum-likelihood tree was constructed using MEGA X (Kumar et al. 2018) under the Tamura-Nei model (Tamura and Nei 1993).

The newly determined complete mitogenome of *B. hamata* (GenBank accession number MW528029) is 17,561 bp in length, containing 12 protein-coding genes (PCGs) (*atp8* gene absent), 23 transfer RNAs (tRNAs), two ribosomal RNAs (rRNAs), and one control region (CR). The overall base composition of Korean *B. hamata* is 29.3% A, 24.1% C, 15.2% G, and 31.4% T, revealing a high A + T content (60.7%). The gene arrangement of the 12 PCGs (*cox1*-*nad6*-*nad3*-*cytb*- *nad4*-*cox3*-*atp6*-*nad5*-*nad4l*-*cox2*-*nad1*-*nad2*) is unique compared to that of the other polychaetes currently available in GenBank.

To confirm the molecular phylogenetic relationship, the maximum-likelihood tree was constructed based on the concatenated sequences of 11 protein-coding genes (*atp6*, *cox1*, *cox2*, *cox3*, *cytb*, *nad1*, *nad2*, *nad3*, *nad4*, *nad4l*, and *nad5*) from eight polychaetes, including the present sequence. Oligochaete was used as an outgroup (*Lumbricus rubellus*). As a result, *B. hamata* belonging to the family Spionidae was clustered with other sedentary polychaetes and formed monophyly (Figure 1). This supports the traditional taxonomic position of Spionidae within subclass Sedentaria. The newly determined complete mitogenome in the order Spionida will provide useful information for further phylogenetic studies.

**Figure 1.**
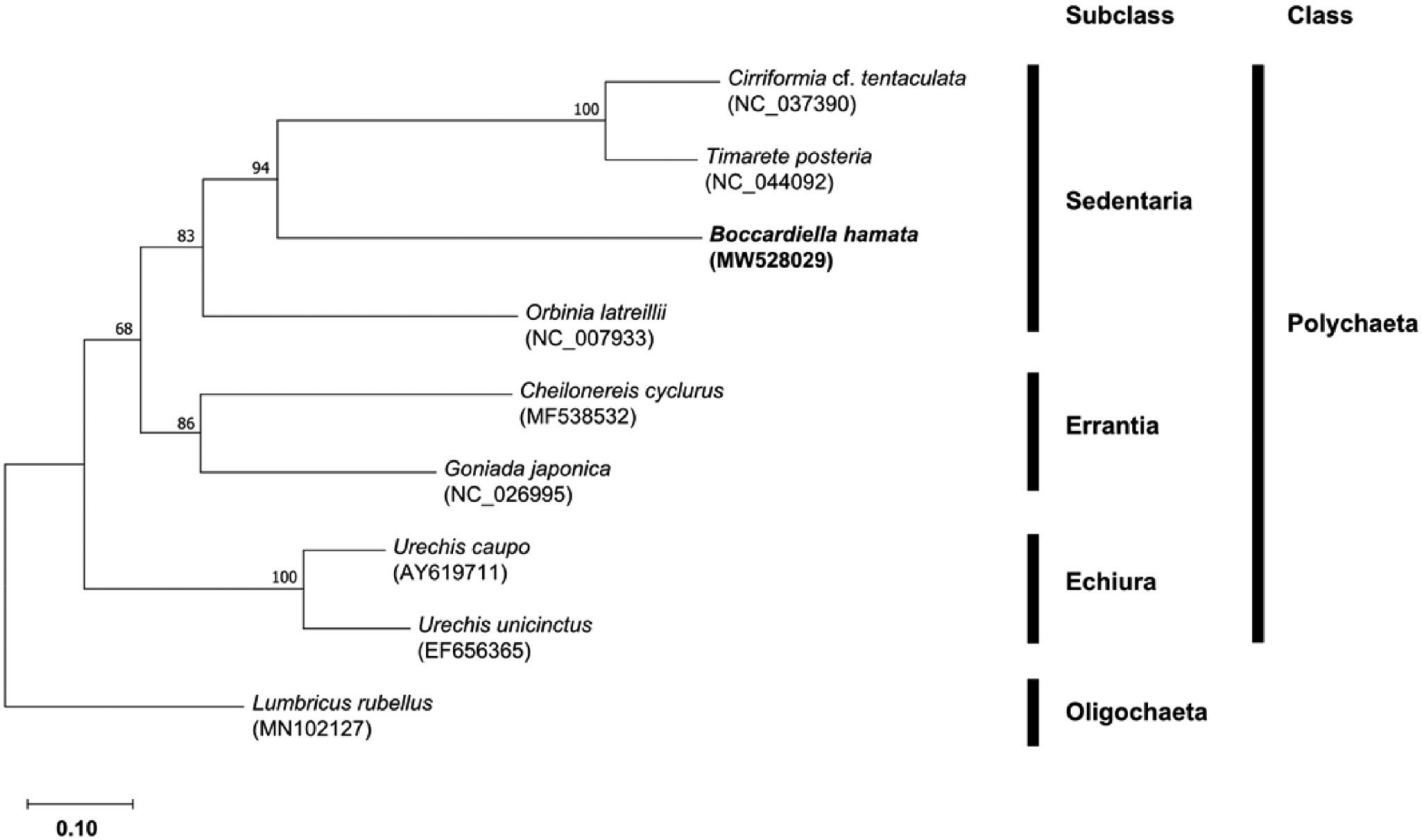
Maximum-likelihood (ML) tree based on the mitogenome sequence of *Boccardiella hamata* (MW528029) with eight polychaete species. *Lumbricus rubellus* was used as an out-group for tree rooting. The bootstrap supports are shown on each node.

## Data Availability

The data that support the findings of this study are openly available in GenBank of NCBI at (https://www.ncbi.nlm.nih.gov/nuccore/MW528029) under the accession no. MW528029. The associated BioProject, SRA, and Bio-Sample numbers are PRJNA748233, SRR15196284, and SAMN20310808, respectively.
